# The Great War: IV. Sickness

**Published:** 1918-03-23

**Authors:** 


### THE GREAT WAR.

### IV.—SICKNESS.

In previous articles we described the wonderful progress which has been made, and continues to be made, in the arrangements for the evacuation and care of the wounded; we will now deal with "sickness."

In a general sense the Expeditionary Force is the healthiest community in the world. It is mainly composed of young men of selected physique, who are well fed and well clothed and who lead a healthy life in the open air under such sanitary conditions as are possible; there is a limitation to this "possible" imposed by military necessities, such as overcrowding, in huts, tents, barns, cellars, dug-outs, and billets; and the exhausting effects of exposure to bad weather, want of sleep, and neglect of personal cleanliness during prolonged tours of duty in the trenches. These unavoidable and irremediable exceptions to healthy conditions are quite sufficient to justify a recurrence of those diseases which destroyed armies in the past, but the long-hoped-for impossible, the miraculous, the inconceivable, has materialised: the scientists have triumphed, for enteric fever and dysentery, those fell diseases which have previously decimated armies in every part of the world, have been eliminated. Enteric is now a negligible factor, and dysentery occurs only under exceptional circumstances.

### Enteric Inoculation the Salvation of an Army.

The most successful Generals in the great war are those Generals of science who have won such a decisive victory over the ubiquitous and elusive enteric bacillus: it has been an uphill struggle of twenty years' duration, marked by adversity, opposition, and accident, but inoculation has at last been universally accepted as essential for the purposes of war. The composition and quality of the vaccine and the method and organisation of its administration are constantly progressing. Quite recently a polyvalent vaccine of enteric and the paratyphoids was adopted, already, we were informed, with the most gratifying results. As an aid in the prevention of enteric fever the search for and segregation of carriers has doubtless played a part. The experiments for ridding carriers of their germs have not been successful; they settle down in their inaccessible home in the gall bladder and defy bombardment by electric rays and the insidious attacks of vaccines. Carriers are not allowed to return to the Army; they are taught how to avoid being a danger to the community and are sent to their homes. Their disposal is one of the problems of the near future.

### Dysentery.

Dysentery has not lived up to its historical reputation. The troops are well fed on the Army ration, supplemented by fresh vegetables and other extras purchased locally, and the water they drink is that drunk by the inhabitants, made doubly safe by sterilisation; consequently their resistance to this disease is high. There was, however, a local outbreak connected with one of our advances into a devastated area, studded with shell craters and pits, full of fouled water, which proved too tempting for thirsty Tommies; the outbreak, however, entirely disappeared from this area on the restoration of a pure water supply.

The local type of the disease is bacillary, but sporadic cases of the amœbic variety, introduced by troops from India and the Mediterranean, crop up from time to time. The spread of the disease is controlled by mobile bacteriological laboratories, which examine every suspected case, and by the segregation of carriers until they are germ-free. A prophylactic vaccine has also been prepared, ready to meet any future requirements.

### Prevailing Diseases are Trivial.

The prevailing diseases in this war are fortunately of a much simpler and milder type. Approximately between 2 and 3 per thousand of strength are admitted to field ambulances daily; this ratio is fairly constant throughout the different seasons of the year, but there is from time to time a local rise due to some special cause such as, for instance, trench feet, or measles. The commonest diseases are trench fever, local septic diseases, including boils and scabies.

Trench fever is accountable for about 1 per thousand of strength, skin diseases, boils, and scabies for about 1 per thousand, and a varied assortment of ailments, such as bronchial catarrh, sore throat, digestive complaints, myalgia, dental troubles, hernia, and sporadic cases of infectious diseases, for the remaining ½ per thousand.

### Trench Fever.

Trench fever is a new disease peculiar to this war. Two clinical types of the disease are distinguished, the one a short fever of a few days' duration followed in four to five days by a single relapse, and the other a longer illness characterised by the number, sharpness, and periodicity of the relapses. Headache behind the eyes and "trench shins" are the only constant symptoms.

Convalescence is rapid in the strong, but the war-weary require six weeks' rest and a change of scene. Its etiology has not yet been determined; it is an intracorpuscular poison, for the transfusion of red corpuscles from the infected into the healthy is followed by an attack on the eighth day. Several kinds of germs have been discovered in various tissues and organs of the infected—spirochætes, cocci of various forms, and bacilli—but the causation still remains in the realms of research.

Reliable statistics prove that the disease is contracted in equal numbers by those in the trenches

☛ Previous articles appeared on Feb. 9 and 23, and March 9, p. 479.

**THE GREAT WAR**—*(continued)*.

and those in rest billets near the trenches; very few cases originate behind the fighting area. The louse is under strong suspicion as an intermediary, but cases occur as far back as Corps Headquarters under circumstances which preclude that origin; there may, however, be more than one mode of dissemination.

"I.C.T."

I.C.T. stands for "Inflammation of connective tissues"; it covers a multitude of minor local septic infections, including boils and the effects of vermin, all due to the difficulties in maintaining personal cleanliness.

Scabies.

In spite of strenuous efforts to grapple with this annoying complaint the "constantly under treatment" rarely sink below .2 per cent. of strength of front-line troops, and the rate rises to as much as .75 per cent. during long spells of duty in the trenches. Its early detection and segregation and the constant disinfection of blankets and clothing are successfully employed in its prevention when the military situation permits, but during active operations opportunity is lacking and the acarus prevails. The disease in approximately half the number of cases is complicated by impetigo and other septic infections which delay treatment: the worst cases of this type eventually find their way to the special skin hospitals at the bases.

Trench Foot.

Much of the area of operations in France is waterlogged, more especially in the northern parts; the subsoil water even in the summer in many of the sectors of the line does not sink below three feet, and during the winter months it rises to ground level or above it. The soil is mostly alluvium mixed with clay, rarely there is chalk or more rarely sand. No strain of the imagination is therefore required to picture the conditions of traffic during the winter rainy season; any deviation from the metalled roads or duck-boarded paths results, in the case of wheeled transport to submersion to the axle-trees, and in the case of pedestrian traffic, to a gradual sinking in tenacious gluey mud to any extent up to the thigh.

The duties of the soldier do not at all times enable him to confine his walks and sojourns to paved roads and duck-boards; the walls of trenches fall in, and duck-boards are buried, the ground water may be so near the surface that trenches cannot be dug, and breastworks take their place, rims of muddy shell-craters also may form the defensive line, all conditions which are conducive to wet feet; and cold, wet feet endured for a sufficient length of time cause "trench feet."

Danger of Unpreparedness.

Trench foot is capable of destroying an army in a very short time. Preventive measures are equally sure of saving an army. As each successive winter comes round, timely warnings and instructions are circulated, which, as experience is gained, constantly become more effective. It is difficult, however, for those who have had no previous experience of a winter on the Western Front to realise on a fine August or September day what is so soon to fall upon the unprepared, and October generally produces some surprises; then the efficacy of preventive organisation is most convincing, for the casualties respond to it by a fall even more rapid than the rise.

Dry Feet.

The remedy, to the unimaginative, presents no difficulties, it is simply the maintenance of dry feet; but in practice it involves one of the most intricate problems in organisation connected with the war, and one which demands the meticulous co-ordination of every branch of the Army—the General Staff, who must adapt the movements of the troops not only to the military situation but to the amount and temperature of the mud; the Adjutant and Quartermaster-General's branch with the technical staffs; the Royal Engineers, who assist the troops in providing dry approaches to, and dry standings in, the trenches by digging, draining, pumping, and revetting; the Army Service Corps, who provide the extra rations of Oxo, cocoa, or tea for the night; the Medical Services, who devise and supervise the methods of prevention; the Ordnance Corps, who provide gum boots and socks; and, lastly, the company and platoon officers who are on the spot, and are primarily responsible that the measures are actually carried out.

Whale Oil and Warm Feet.

During the trench-foot season, before starting on his tour of duty in the trenches each soldier has his feet washed and rubbed until they are warm, and whale oil or anti-frostbite grease well rubbed into them. This application results in the comparative conservation of heat to the extent of seven degrees Fahrenheit.

Dry Socks and Gum Boots.

The next duty is to put on dry socks and to receive a spare pair, sometimes two pairs, for future use. Then the question of boots arises. Gum boots are better for wet trenches and leather ones for dry ones, irrespective of temperature. The air spaces in leather boots are non-conductors, and conserve heat; they also allow a useful amount of transpiration. Gum boots confine the perspiration, the feet and socks become sodden, especially after a long walk, and heat is gradually lost to the air more slowly in water. In principle, therefore, leather boots are worn in dry trenches and gum boots in wet ones, and they are put on as near the commencement of the wet as possible. The drying of gum boots is quite an undertaking. Extensive drying-rooms are established under brigade arrangements to which boots are delivered as the men return from the trenches. There as much water as possible is removed by cloths; they are suspended upside down to drain, and the drying is finished in the upright position, the whole process taking twenty-four hours. A clean, dry pair of socks per man is delivered daily at the trenches by the ration party in exchange for a used pair; these are changed and the feet rubbed and oiled in appointed dug-outs in or near the firing-line.

Hot Drinks, Exercise, Frequent Reliefs.

Other preventive measures are the provision of hot soup, cocoa, or tea during the night, and arrangements for keeping every man exercised. The question of relief is also essential; it is not safe to keep men in cold, wet trenches for longer than four days, and in exceptional cases the tour is reduced to twenty-four hours. The system is controlled by a detailed report on each case, which travels from the platoon officer through the company, battalion, brigade, divisional, and corps headquarters to the respective armies. With so many "Brass Hats" eagerly on the watch to render assistance the intense interest in the subject taken by the battalion officers is not surprising, nor are the results disappointing.

(*To be continued.*)

